# The lncRNA 44s2 Study Applicability to the Design of 45-55 Exon Skipping Therapeutic Strategy for DMD

**DOI:** 10.3390/biomedicines9020219

**Published:** 2021-02-20

**Authors:** Elena Gargaun, Sestina Falcone, Guilhem Solé, Julien Durigneux, Andoni Urtizberea, Jean Marie Cuisset, Sofia Benkhelifa-Ziyyat, Laura Julien, Anne Boland, Florian Sandron, Vincent Meyer, Jean François Deleuze, David Salgado, Jean-Pierre Desvignes, Christophe Béroud, Anatole Chessel, Alexia Blesius, Martin Krahn, Nicolas Levy, France Leturcq, France Pietri-Rouxel

**Affiliations:** 1 SU, INSERM UMRS974, AIM, Center of Research in Myology, Pitié-Salpêtrière Hospital, 75013 Paris, France; s.falcone@institut-myologie.org (S.F.); s.ziyyat@institut-myologie.org (S.B.-Z.); laura.julien003@gmail.com (L.J.); france.pietri-rouxel@upmc.fr (F.P.-R.); 2 Centre de Référence des Maladies Neuromusculaires AOC, Service de Neuropédiatrie, CHU Bordeaux, 33000 Bordeaux, France; guilhem.sole@chu-bordeaux.fr; 3 Centre de Référence des Maladies Neuromusculaires AOC, CHU Angers, 49933 Angers, France; JuDurigneux@chu-angers.fr; 4 Institute of Myology, Hôpital Pitié-Salpêtrière, 75013 Paris, France; ja.urtizberea@free.fr; 5 Centre de Référence des Maladies Neuromusculaires Nord/Est/Ile de France, Service de Médecine Physique et de Réadaptation, CHRU de Lille, 59000 Lille, France; jm-cuisset@chru-lille.fr; 6 CEA, Centre National de Recherche en Génomique Humaine, Université Paris-Saclay, 91057 Evry, France; boland@cng.fr (A.B.); sandron@cng.fr (F.S.); meyer@cng.fr (V.M.); deleuze@cng.fr (J.F.D.); 7 INSERM, Marseille Medical Genetics, Aix Marseille University, 13005 Marseille, France; david.salgado@univ-amu.fr (D.S.); jean-pierre.desvignes@univ-amu.fr (J.-P.D.); christophe.beroud@inserm.fr (C.B.); martin.krahn@univ-amu.fr (M.K.); nicolas.levy@univ-amu.fr (N.L.); 8 Département de Génétique Médicale, APHM, Hôpital d’Enfants de la Timone, 13005 Marseille, France; 9 Laboratoire d’Optiques et Biosciences (LOB), CNRS, INSERM, École polytechnique, Institut Polytechnique de Paris, 91120 Palaiseau, France; anatole.chessel@polytechnique.edu; 10 IRIS, Institut de Recherches Internationales Servier, 92150 Suresnes, France; alexia.blesius@servier.com; 11 AP-HP, Laboratoire de génétique et biologie moléculaires, Hôpital Cochin, Université Paris Descartes-Sorbonne Paris Cité, 75014 Paris, France; France.leturcq@inserm.fr

**Keywords:** long noncoding RNA, lncRNA, ncRNA, Becker muscular dystrophy (BMD), Duchenne muscular dystrophy (DMD)

## Abstract

In skeletal muscle, long noncoding RNAs (lncRNAs) are involved in dystrophin protein stabilization but also in the regulation of myocytes proliferation and differentiation. Hence, they could represent promising therapeutic targets and/or biomarkers for Duchenne and Becker muscular dystrophy (DMD/BMD). DMD and BMD are X-linked myopathies characterized by a progressive muscular dystrophy with or without dilatative cardiomyopathy. Two-thirds of *DMD* gene mutations are represented by deletions, and 63% of patients carrying *DMD* deletions are eligible for 45 to 55 multi-exons skipping (MES), becoming BMD patients (BMDΔ45-55). We analyzed the genomic lncRNA presence in 38 BMDΔ45-55 patients and characterized the lncRNA localized in introns 44 and 55 of the *DMD* gene. We highlighted that all four lncRNA are differentially expressed during myogenesis in immortalized and primary human myoblasts. In addition, the lncRNA44s2 was pointed out as a possible accelerator of differentiation. Interestingly, lncRNA44s expression was associated with a favorable clinical phenotype. These findings suggest that lncRNA44s2 could be involved in muscle differentiation process and become a potential disease progression biomarker. Based on these results, we support MES45-55 therapy and propose that the design of the CRISPR/Cas9 MES45-55 assay consider the lncRNA sequences bordering the exonic 45 to 55 deletion.

## 1. Introduction

Skeletal muscle is the principal actor of human body movement and posture maintenance. The genesis of the skeletal muscle—myogenesis—encounters several distinct phases [1,2]. Each step of myogenesis is orchestrated by complex intrinsic and extrinsic regulators, including the myogenic regulatory factors (MRF), a group of highly conserved genes expressed in skeletal muscle lineage: Myf5, myogenic differentiation antigen-MyoD, myogenin-MyoG, myogenic regulatory factor MRF-4, myosin heavy chain MHC [3,4]. The MRF, together with the Paired box (Pax) family members Pax3 and Pax7, act at hierarchically defined timepoints to regulate cell cycle, proliferation, and differentiation [5,6]. Moreover, previous works underline that common progenitor cells maintain muscle growth during late embryonic development and assure adult muscle growth and repair [6,7,8].

Along with the multiple myogenic regulatory factors, long noncoding RNA (lncRNA), a class of noncoding RNA > 200 nucleotides in length with no protein coding function, emerge as new key role players [9,10,11,12]. Recent high-throughput RNA sequencing (RNAseq) experiments identified lncRNA that are differentially expressed and involved in myogenesis [13,14]. For example, lncRNA SRA (steroid receptor RNA activator) associates with p68/p72 and acts as transcriptional coactivators of MyoD [15]. Moreover, SRA was found to have a particular feature as both noncoding RNA and protein (SRAP, because its expression follows an alternative splicing event). The noncoding SRA transcript is abundantly expressed in differentiating myoblasts and participates in MyoD activation. Conversely, the protein SRAP physically binds to this transcript homolog SRA, preventing MyoD activation and, consequently, myogenic differentiation [16]. Linc-MD1, another lncRNA, was found to be abundantly expressed during differentiation in myotubes and, interestingly, in newly regenerating muscle fibers [17]. In addition, bioinformatic studies revealed that binding sites for miR-133 and miR-135 were conserved in Linc-MD1 sequence and reported its association with these muscle-specific miRs as a competing endogenous RNA (ceRNA) [18]. In addition, it has been described that Linc-MD1 sequesters miR-133 or miR-135 as a sponge, inhibiting the binding of these miR to transcription factors such as MEF2C and MAML1, required for myogenesis [17]. Furthermore, linc-MD has been demonstrated to bind HuR, a specific RNA binding protein that is under the repressive control of miR-13, creating a reinforced sponging activity [19]. All this evidence pointed out LncRNA and miRs as regulatory players of myogenesis. These features could be important in the pathophysiology of different myopathies, including in Becker and Duchenne muscular dystrophies (BMD/DMD) since alterations in the regenerative processes may influence the dystrophic disease progression and patient outcome.

Both BMD/DMD are X-linked allelic disorders caused by mutations in the *DMD* gene [20,21]. DMD is characterized by mutations leading to mRNA transcription interruption, which results in the absence of dystrophin protein expression and severe muscular deficit-inducing loss of ambulation (LoA) in young boys and shortened life span. In contrast, in BMD disease, mRNA open reading frame is maintained, resulting in the production of a shortened but functional dystrophin, which leads to a less severe phenotype. Due to this exceptional particularity, the therapeutic dogma is to convert DMD into milder BMD phenotype by altering the pre-mRNA splicing through exon skipping approaches in order to restore the open reading frame, allowing the translation of an internally deleted and partially functional protein [22,23,24]. As of today, competent health authorities (both FDA/EMA) have approved antisense oligonucleotides (AONs) such as exon skipping 51 (EXONDYS 51, Sarepta, Cambridge, MA, USA) and exon skipping 53 (VYONDYS 53 Sarepta, Cambridge, MA, USA), which cover approximatively 10% and 13% of patient deletion mutations, respectively. The multiple exon skipping (MES) of exons 45 to 55 could theoretically enlarge DMD patient eligibility to such therapeutic approaches as antisense oligonucleotides (AON) and CRISPR-Cas9-based therapeutic strategies by up to 63% of deletion mutation patients. Thereby, the nonhomologous end-junction (NHEJ) reframing of DMD 45-55 exons in immortalized DMD patient myoblasts (ex.48-50deletion) lead to successful dystrophin rescue [25,26,27].

In our previous study, we underlined the phenotype variability in BMD patients with exon 45-55 deletion (BMDΔ45-55) [28]. Here we report breakpoint analysis by whole genome sequencing (WGS), performed in 18 patients of a BMDΔ45-55 population, and show the specific breakpoints involved the deletion of the lncRNA sequences. To go further, we analyzed the presence of lncRNA bordering the DMD 45-55 deletion in 38 BMDΔ45-55 patients and examined their skeletal muscle expression. The lncRNA 44s and 44s2 expression pattern was studied in skeletal muscle biopsy of control, BMDΔ45-55 patients, BMDΔ3-7 patients, and BMDΔ45-55. The profile of expression of these lncRNA in dystrophic myoblasts issued from BMDΔ45-55(45-55) and DMDΔ45-52(45-52) patients confirmed what was observed in skeletal muscle biopsies. Furthermore, overexpression of the ncRNA44s2 revealed its role in the skeletal muscle regeneration mechanism.

Altogether, these results raise the question of the correlation between the favorable clinical phenotype of BMDΔ45-55 and the lncRNAs in intron 44.

## 2. Materials and Methods

### 2.1. Ethic Statement

This study was reviewed and approved by the Cochin Hospital Institutional Review Board. Written consent was obtained for diagnostic and research purposes. All participants were assigned de-identified numbers. With signed informed consent, diagnostic muscle biopsies were collected from BMD and DMD patients and healthy controls.

### 2.2. Population Characterization

The cohort included male BMD patients with an exclusive deletion mutation of exons 45 to 55 whatever their age. The population was taken from the French UMD-DMD database cohort identified in Cochin hospital [29]. The phenotype characterization was performed retrospectively from the medical records.

Retrospectively collected phenotype data included skeletal muscle symptoms and/or deficit (muscle weakness or wasting, calf hypertrophy, fatigability, myalgia, exercise, and/or effort intolerance, etc.) and age at first symptom appearance (if present), age at first skeletal muscle signs, age at last clinical examination, age at diagnosis, report/last follow-up (if available), loss of ambulation (LoA) and age at LoA, cardiomyopathy (history, age at diagnosis, first and last available left ventricular ejection fraction, respiratory involvement (history, noninvasive ventilation if relevant and the age at initiation when available), biopsy (history, result of immunofluorescence and Western blot), and creatinine kinase blood level at initial and last available evaluation. Cognitive evaluation status in adult patients (>18 y.o.) was considered as normal if no specific comments were found in the medical file.

### 2.3. Modified Clinical Severity Scale (CSS)

In order to assess skeletal muscle impairment, we used the previously reported clinical characterization and, given the favorable phenotype of this cohort, modified the assignment criteria for clinical status [30]. We decided to make 3 categories of skeletal muscle complaints: in the first category were included all the symptoms except muscular deficit, such as cramps, myalgia, fatigue, effort and/or exercise intolerance, episodes of myoglobinuria and elevated isolated creatine kinase (CK). In the second category were included patients with skeletal muscle deficit, and in the third category were included the patients who lost ambulation (LoA). The score rules were as follows: if there were no complaints (type 4), the patient was classified as asymptomatic BMD (ABMD); the presence of only symptoms was evaluated by the practitioner as type 3, mild BMD (MBMD); if the patient reported muscular deficit alone or presented with symptoms, he was assigned into the intermediate type 2 (IBMD); and in the severe type 1 (SBMD) were the patients who lost partial or total ambulation. All patients with available clinical data in order to perform the CSS scoring were assigned into a specific type; in order to address the disease progression evaluation, we excluded from the CSS scoring the patients < 20 years of age, as we considered that there would not be enough information about disease progression.

### 2.4. Cell Culture

Isolated human primary myoblasts from healthy subjects and DMDΔ45-52 deletion patients were kindly provided by Pr. F. Muntoni (King’s College, London, UK), and the BMDΔ45-55 human primary myoblasts were provided by the Dr. N. Streichenberger (HCL Lyon, France). The human immortalized cell lines (45-52 and Hthy) were provided by Dr. V. Mouly, Dr. K. Mamchaoui, and Dr. A. Bigot (MyoLine facility Institute of Myology, Paris, France).

For proliferation, both primary and immortalized myoblasts were maintained in skeletal muscle cell growth medium (DMEM supplemented with 5 µg/mL of insulin, 5 ng/mL of EGF, 0.5 ng/mL of bFGF, 0.2 µg/mL of dexamethasone only for immortalized cell lines, 25 µg/mL of fetuin, 20% of fetal bovine serum, and 16% of medium 199). For differentiation experiments, primary and immortalized myoblasts were maintained in DMEM supplemented with 2% horse serum. Proliferation and differentiation for both primary and immortalized cell lines were supplemented with 50 µg/mL of gentamycin.

### 2.5. DNA/RNA Purification and Analysis

Healthy control and BMD and DMD patient DNA extracted from diagnostic blood samples were analyzed by a DNA kit (Macherey-Nagel, Gmbh & Co KG, Duren, Germany). Total RNA of skeletal muscle biopsies and human primary and immortalized myoblasts from a healthy subject (Hthy) and from a DMDΔ45-52 patient (DMDΔ45-52) and BMDΔ45-55 patient (DMDΔ45-55) extractions were performed using Nucleospin miRNA kit (Macherey-Nagel, Gmbh & Co KG, Duren, Germany). Reverse transcription (RT) was performed on 250 ng of mRNA by using Superscript II and random hexamers (Life Technologies AS, Oslo, Norway). RT-PCR products were separated by electrophoresis on 3% Agarosis gel with ethidium bromide. qPCR was performed on the StepOnePlus ^TM^, ThermoFisher Scientific, Waltham, MA, USA) system with SYBR Green/TaqMan reagent using primers shown in Table S1. Relative expression between proliferating and differentiated myoblasts and transduced and control myoblasts was measured in duplicates within 4 separate experiences. Statistical significance was analyzed using two-tailed Student’s test and ANOVA on GraphPad Prism.

### 2.6. Adeno-Associated Virus Vector (AAV) Production and lncRNA Overexpression Experiments (OE)

The lncRNA44s2 cDNA sequence flanked by restriction enzyme sites (MluI, XhoI) was directly cloned in pSMD2 AAV9 vectors backbones, under CMV promoter (GeneArt string; ThermoFisher Scientific, Waltham, MA, USA). The final viral preparations were kept in PBS solution at −80 °C. The particle titer (number of viral genomes) was determined by quantitative PCR. The proliferation and differentiation medium were specified earlier.

For the proliferation experiments, the human immortalized and primary myoblasts from control, BMDΔ45-55, and DMDΔ45-52 patients were plated 24 h before transduction (D0) 3.5 × 10^5^ in a 24-well plate in proliferation medium. At D1, myoblasts were transduced at MOI (multiplicity of infection) (3.5 × 10^5^) in 300 μL with AAV-nc44s2, AAV-Scramble-GFP, and transduction medium for the healthy type (ctr) condition. Cells were counted on the Acova plate and the cell platelet was collected at the third day of proliferation (P3).

For the differentiation experiments, the human immortalized and primary myoblasts from control, BMDΔ45-55, and DMDΔ45-52 patients were plated 24 h before transduction (D0) 5.0 × 10^5^ in a 24-well plate until confluent (80% of cell confluence) and then switched to differentiation medium. At 80% of confluence, the myoblasts were transduced at MOI 3.5 × 10^5^ in 300 μL with AAV-nc44s2, AAV-Scramble-GFP. In the healthy condition, the proliferating medium was changed to differentiation medium at 80% of confluence. Cell platelet was collected at the third day of differentiation (D3). The fusion index was evaluated by myotubes nuclei count/total nuclei number in at least three representative images.

### 2.7. Immunohistochemistry

Primary and immortalized human myotubes were fixed with paraformaldehyde 4% 10 min at room temperature (RT) and washed 3 times in PBS. Fixed cells were permeabilized with 0.5%Triton X-100/PBS (Sigma-Aldrich, Darmstadt, Germany) for 10 min at RT and blocked with bovine serum albumin (BSA) 4% for 45 min at RT. Primary hybridoma antibody MF20 (Institute of Myology) was applied overnight at 4 °C, washed, and visualized with fluorochrome-conjugated secondary antibody donkey anti-goat (Alexafluor 594 conjugate, (Life Technologies, AS, Oslo, Norway) 1:500) for 1 h at RT. Fixed cells were washed in PBS/1%, BSA/0.1%, saponin, and then in PBS for 10 min. Images were analyzed with EVOS (ThermoFisher Scientific, Waltham, MA, USA ) microscope using a x10/x20 objective.

### 2.8. Bioinformatic Analysis

Whole genome sequencing (WGS) was performed on 1 µg of purified genomic DNA of 18 patients by Illumina sequencing (San Diego, CA, USA) using Illumina TruSeq DNA PCR-Free library preparation kits and the Illumina HiSeqX5 sequencing platform of the CNRGH.

The annotation of 18 genomes was performed by VarAFT annotation and filter for the SNP (single nucleotide polymorphism)/INDELS (insertion-deletion mutations). VarAFTv2.14 provided experiment quality, annotation, and filtration of VCF files [31]. DNA genomic breakpoint analysis was performed by the BreakDancer [32], Control-FREEC [33], and Delly 2 [34] software in order to identify the deletions. Then, manual identification of precise breakpoint (±10 base pairs (bp) precision) was performed using each sample binary alignment map (BAM) files with the Integrative Genomics Viewer ( IGV_2.4.10) tool [35].

## 3. Results

### 3.1. BMDΔ45-55 Patients lncRNA Breakpoints Impact in lncRNA Sequences

**Figure 1:**
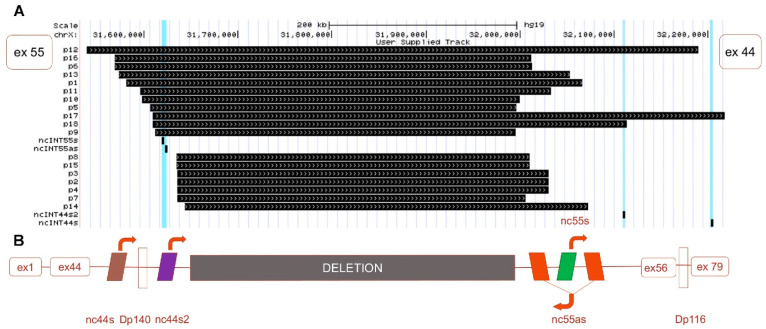
Neo-intron of deleted 45-55 exons in Duchenne muscular dystrophy (DMD) gene. (**A**) Schematic representation of the whole genome sequencing (WGS) detection of the deletion breakpoint (black boxes) in the DMD gene of 18 patients BMDΔ45-55. (**B**) Schematic representation of the canonic exon 45-55 deletion neo-intron with the lncRNA (colored boxes for lncRNA and arrows to indicate the transcription sense. The exons 1 to 44 and 56 to 79 are illustrated in clear red boxes).

Our previous works underlined a clinical phenotype variability in a cohort of BMDΔ45-55 and demonstrated the involvement of miR-708 and miR-34c in the regulation of *nNOS* expression, and therefore in the pathophysiology of BMD in these patients [28,36]. The present study concerns the question of the regulatory mechanisms in the phenotype variability in BMDΔ45-55 patients. To determine the precise coordinates of the deletion, a WGS analysis was carried out in diagnostic DNA of 18 patients. Based on WGS data, we determined that, with the exception of family cases, each patient’s *DMD* sequence displayed a specific deletion breakpoint (Figure 1). Then, the impact of the specific breakpoint on the lncRNA sequence present in the patient’s *DMD* gene was investigated.

**Figure 2:**
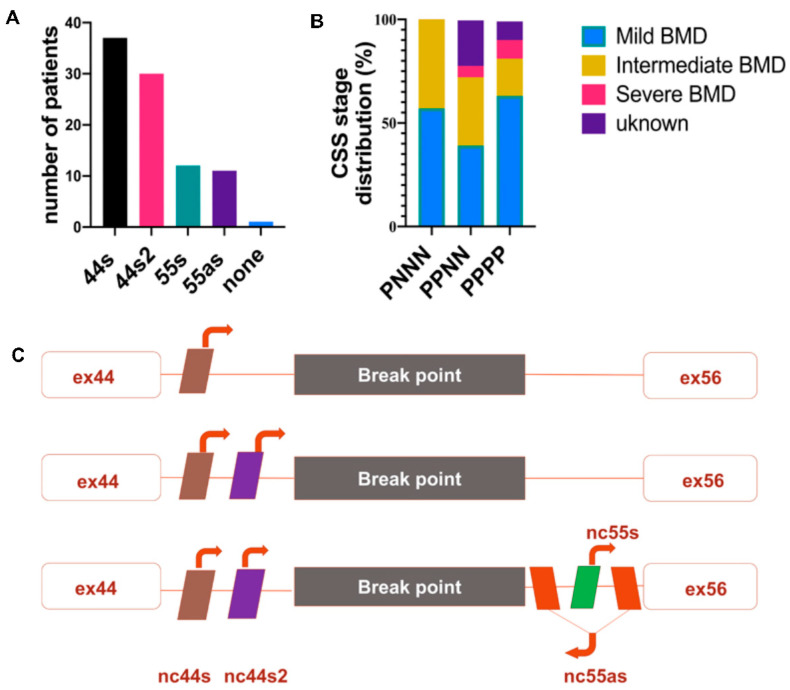
lncRNA profile in BMDΔ45-55 patients (*n* = 38). (**A**) lncRNA detection by PCR from diagnostic DNA, the genomic presence of each lncRNA was annotated by P and the absence by N. (**B**) Scheme presentation of the most frequent lncRNA genomic presence profiles. (**C**) Phenotype correlation with lncRNA genomic clusters. Abbreviations: P = present lncRNA sequence, N = absent lncRNA sequence, MBMD = Mild Becker muscular dystrophy (BMD), IBMD = Intermediate BMD, SBMD = Severe BMD, ex = exon).

Only one BMDΔ45-55 patient displayed a large deletion in *DMD* gene that involved all lncRNA sequences (i.e., 2% of the tested patient cohort); in 7 patients, the lncRNA 44s2, 55s, and 55as were deleted (18%); in 18 patients, the deletion breakpoint removed only the lncRNA localized in intron 55 (47%); in 11 patients (29%), the deletion conserved all the lncRNAs; and in one patient (2%), conserved lncRNA 44s, 44s2, 55s (Figure 1A). Overall, we report a major trend of intron 44 lncRNA conservation and a second one conserving all lncRNAs. To confirm this observation, breakpoint profiling was completed by the detection of lncRNA sequences bordering the exonic deletion in a total of 38 patients on DNA samples extracted from diagnostic blood samples. These data highlighted that the lncRNA 44s and 44s2 were spared by the deletion in the majority of the BMDΔ45-55 patients (*n* = 30, 79%) (Figure 2A). Correlation with clinical phenotype revealed a trend to less severe skeletal muscle deficit phenotype in patients with the conserved lncRNA44s (Figure 2B). The age at which first skeletal muscle signs appeared in the intermediate patients included in the PNNN cluster was 43 ± 5 years old and in the PPNN cluster was 14 ± 16 years old. The age at last examination for patients included in the PNNN cluster was 46 ± 10 years old and in the PPNN cluster was 27 ± 24 years old, respectively. In addition to an earlier age featuring skeletal muscle deficit, the intermediate phenotype patients from the PPNN cluster had more severe symptoms then those in PNNN clusters.

Despite the increase of the patients’ number, all lncRNA sequences were deleted only in one patient. These results revealed the two most frequent deletion breakpoints, one preserving the noncoding sequences localized in the intron 44 and a second one conserving all the lncRNA both from intron 44 and intron 56 (Figure 2C).

### 3.2. lncRNA Expression BMD/DMD Patients’ Skeletal Muscle Biopsies

**Figure 3:**
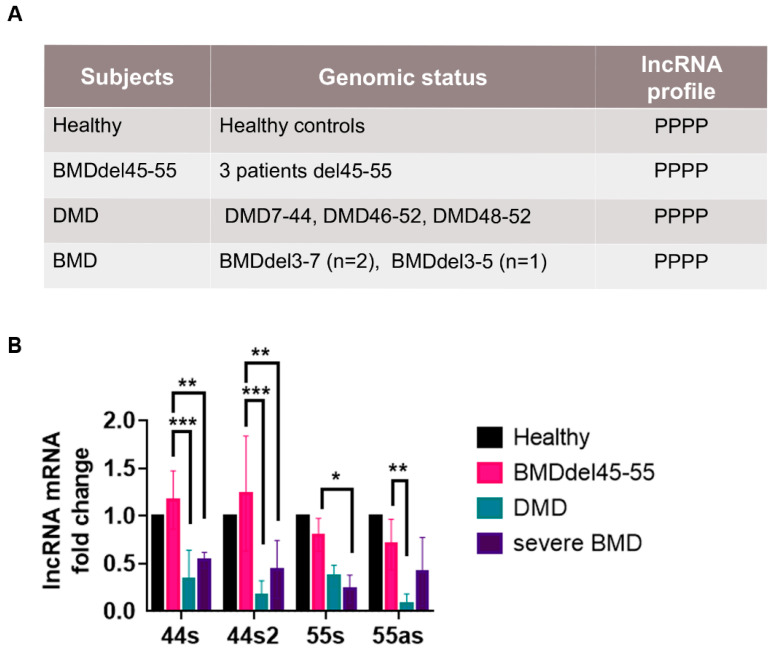
lncRNA profile in control and DMD/BMD patients. (**A**) Genomic profile of control, DMD/BMD patient selected for the expression analysis in muscular biopsy. The genomic presence of each lncRNA was annotated by P. (**B**) Assessment of relative lncRNA expression in the muscular biopsies of a healthy subject and DMD and BMD patients by RT-qPCR (Mean ± SD); lncRNA from the healthy subject was the control and set to 1 (respective *p*-values are indicated * *p* < 0.05, 44s2: ** *p* = 0.001, *** *p* < 0.0005) by two-way ANOVA test. Abbreviations: DMD = Duchenne muscular dystrophy, BMD = Becker muscular dystrophy.

To explore lncRNA expression, a relative quantification by RT-qPCR was performed in muscular biopsies obtained in three BMDΔ45-55, two BMDΔ3-7, one BMDΔ3-5 patient, and three DMD patients (DMD8 = Δ7-44, DMD14 = Δl46-52, DMD7 = Δ48-52) (Figure 3). Indeed, to investigate of the potential impact of the spectrin repeat deleted region on the lncRNA expression levels, several BMD patients with different in-frame deletion mutations were selected.

For the DMD patients, the choice of the mutation was influenced by the available muscular biopsies and also by the exon skipping eligibility of the mutations. Thus, patient DMDΔ7-44 would be eligible to exon 45 skipping therapy, patient DMDΔ46-52 to MES 45-55, and patient DMDΔ48-52 to exon skipping 53 therapy.

First, the lncRNA expression analysis showed that all the lncRNA were expressed in all control subjects’ biopsies. Secondly, the BMDΔ45-55 patients had a similar expression level of all lncRNA compared to controls. Interestingly, all BMDΔ45-55 patients’ lncRNA expression levels were higher compared to the lncRNA expression in BMDΔ3-5 and BMDΔ3-7 patients (lncRNA 44s/44s2: *p* < 0.005; 55s: *p* < 0.001). Finally, the lncRNA expression in DMD patients was lower compared to BMDΔ45-55 patients (lncRNA 44s/44s2: *p* < 0.0005; 55as: *p* < 0.001).

### 3.3. Low lncRNA Expression during Proliferation in Dystrophic Human Myoblasts

**Figure 4:**
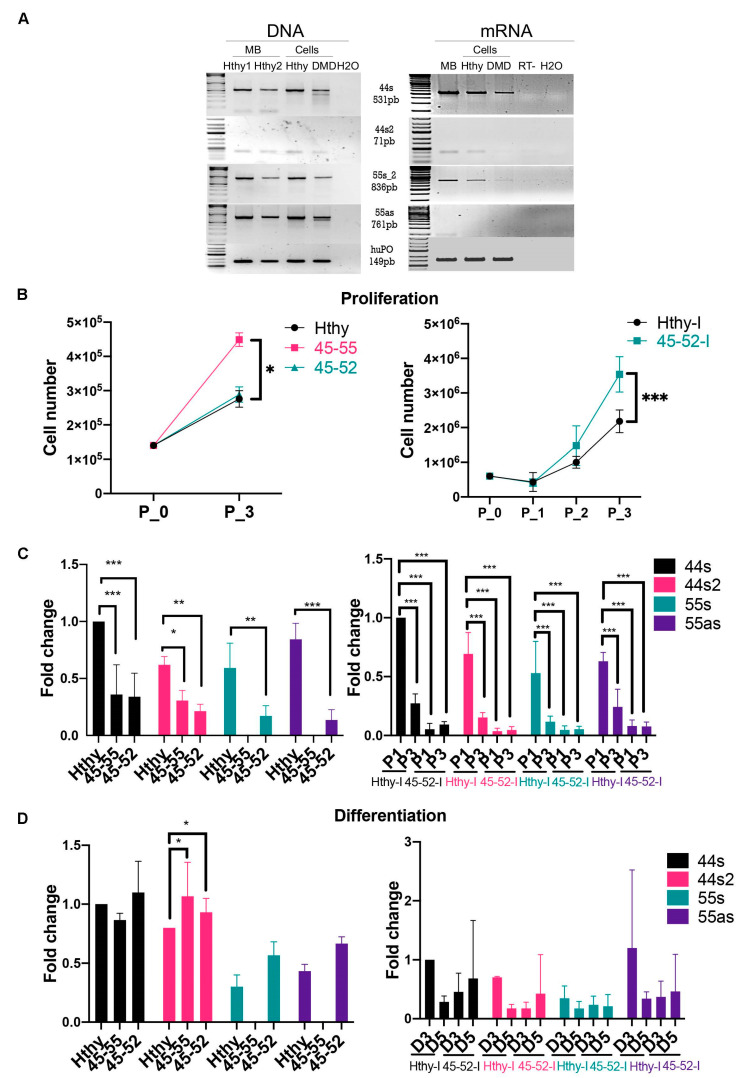
lncRNA genomic profile and expression assessment in human myoblasts. (**A**) DNA and RNA profiling in muscular biopsy (MB) from healthy subject and immortalized human myoblasts from healthy subject (Hthy) and DMDΔ45-52 deletion patient (DMD). (**B**) Proliferation assessment at different timelines (day 0 to day 3/P0-P3) in human immortalized (healthy subject = Hthy-I, DMDΔ45-52 patient = 45-52-I) and primary human myoblasts from healthy subject (Hthy), BMDΔ45-55 (45-55), and DMDΔ45-52(45-52) patients. (**C**) lncRNA expression profile at day 1 (P1) and 3 of proliferation(P3) (left) in human primary and (right) in immortalized myoblasts. (**D**) lncRNA expression was analyzed by RT-qPCR at 3 and 5 days (D3, D5) of differentiation (**left**) in human primary and (**right**) in immortalized myoblasts (black columns illustrate nc44s, red columns = nc44s2, green columns = nc55s, violet columns = nc55as). Data represent the average from three independent experiments (Mean ± SD); * *p* < 0.05, ** *p* < 0.01, and *** *p* < 0.001 by two-way ANOVA test).

lncRNA expression analysis in patient muscular biopsies pointed out that lncRNA44s2 was less expressed in patients with severe dystrophic conditions. Therefore, to address the potential involvement of lncRNA44s2 in the dystrophic process, immortalized cell lines from a healthy subject (Hthy-I) and DMDΔ45-52 (45-52-I) patients were analyzed. The presence of the lncRNA sequences was investigated at the genomic level in these two cell lines and in a muscular biopsy (MB) from a healthy control subject. All of the lncRNA sequences were found in the DNA of both cellular lines and muscular biopsies. RT-PCR allowed the mRNA detection of all lncRNA in Hthy-I myoblasts and lncRNA44s2 and 55as were undetectable in 45-52-I cells (Figure 4A).

The profile of the lncRNA expression was then explored during the proliferation process. As preliminary assessment, a comparative analysis of the proliferation rate of the two immortalized cell lines was performed and showed a statistically significant (*p* < 0.0003) higher rate of 45-52-I cells proliferation compared to Hthy-I myoblasts (Figure 4B). Then, lncRNA expression was investigated during proliferation, and the results revealed a significantly weaker expression (*p* < 0.0001) of all lncRNA in 45-52-I compared to control Hthy-I myoblasts (Figure 4C, right panel).

In addition, the relevance of genomic lncRNA sequences was investigated in primary human myoblasts of control, BMDΔ45-55(45-55), and DMDΔ45-52(45-52) patients. In these myoblasts, proliferation rate was shown to be significantly higher (*p* < 0.0001) in 45-55 cells compared to Hthy and 45-52 (Figure 4B, left panel). Consequently, during proliferation, the expression profile of all lncRNA of interest displayed a significantly lower expression (44s (*p* < 0.0001), 44s2 (P1: 45-52/Hthy *p* = 0.01); P3: 45-52/Hthy *p* = 0.019), 55s (45-52/Hthy = *p* = 0.04), 55as (45-52/Hthy = *p* < 0.001)) compared to control myoblasts (Figure 4C, left panel). Interestingly, there was no difference in the expression of lncRNA 44s/44s2 between the 45-52 and 45-55 at day 3 of proliferation (Figure 4C, left panel).

In conclusion, lncRNA expression quantification at three days of proliferation in both immortalized and primary myoblasts revealed a lower expression in dystrophic myogenic cells compared to control. Conversely, dystrophic myoblasts displayed a higher rate of proliferation in in both primary and immortalized myoblasts. In particular, primary 45-55 and immortalized 45-52 myoblasts showed the highest rate of proliferation compared to their respective controls.

### 3.4. Low lncRNA Expression during Differentiation in Human Myoblasts

The lncRNA expression was investigated 3 days after differentiation in immortalized myoblasts and showed a trend to increased expression in Hthy-I compared to 45-52-I cells, but we could not demonstrate a significant difference (*p* = 0.3) between the various conditions due to the elevated variability in cells (Figure, 4D right). Furthermore, three days after differentiation the expression of lncRNA 44s in 45-55 and 45-52 reached a significantly higher (*p* = 0.03) level than control. The nc44s and 44s2 had approximately 4-fold higher expression compared to proliferation assessment. lncRNA 55s and 55as were absent in 45-55 during proliferation and differentiation reflecting the genomic deletion pattern (Figure 4D, left panel).

Based on the results of lncRNA expression profile in skeletal muscle biopsies and in human myoblasts, we explored the possible role of lncRNA44s2 on myogenesis in control and dystrophic muscle cells.

### 3.5. lncRNA44s2 Overexpression Impacts Human Primary Myoblasts Differentiation

**Figure 5:**
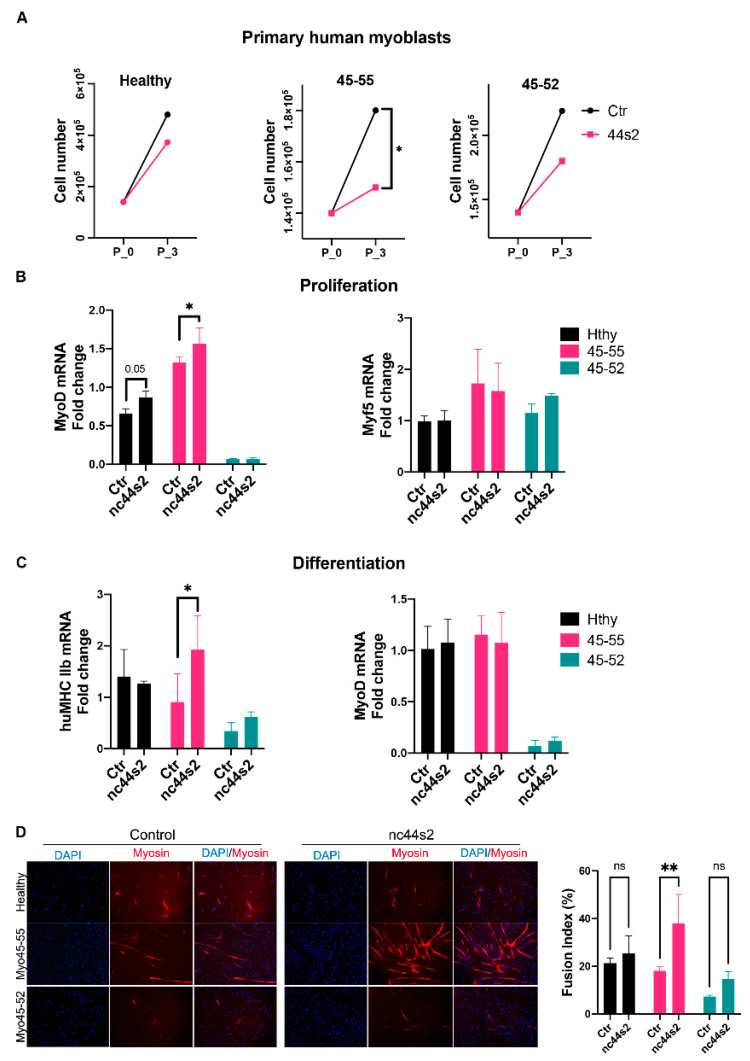
nc44s2 overexpression study during myogenesis in human primary myoblasts. (**A**) Growth charts depicting the proliferation rate in 3 cell lines; red color illustrates the control condition, green the nc44s2 in human primary myoblasts issued from healthy subjects (Hthy), BMDΔ45-55 patients (45-55), and DMDΔ45-52 patient (45-52). (**B**) MyoD and Myf5 expression assessment by RT-qPCR at 3 days of proliferation from three independent experiments performed in duplicates (significant increase in MyoD expression in 45-55 * *p* = 0.02 by two-way ANOVA). (**C**) MyHCIIb and MyoD expression at 3 days of differentiation from three independent experiments performed in duplicates (significant increase in 45-55 of MyHCIIb, ** *p* = 0.03 by two-way ANOVA) (Means ± SD). (**D**) Immunofluorescence experiment illustrating the myotubes at 3 days of differentiation in three primary human myoblasts visualized at X10 magnification. The fusion index was evaluated by myotubes nuclei count/total nuclei number and compared between OE and control conditions in at least three representative images at three days of differentiation (** *p* < 0.004 by two-way ANOVA) (Means ± SD).

The lnc44s2 overexpression (OE) was analyzed during proliferation by RT-qPCR, and data confirmed a significantly higher expression of lnc44s2 in all three cell types (*p* = 0.0001) (Figure S1A). During differentiation, the lnc44s2 OE analyzed by RT-qPCR was significantly increased in the 45-55 myoblasts (*p* = 0.04) but did not reach a significant increase in Hthy and 45-52 cell lines (Figure S1B). The lncRNA44s2 OE induced a decrease of proliferative rate in all three cell lines between nc44s2 conditions compared to control myoblasts (Figure 5A). In order to determine the regulatory role of lncRNA 44s2 during proliferation, myomarkers expression was investigated at three days of proliferation. lncRNA expression profile did not change during proliferation, and the Myf5 expression was not significantly different between control and OEnc44s2 condition (Figure 5B). However, the expression of MyoD was significantly increased in the treated nc44s2 condition compared to control in the 45-55 cell line (*p* = 0.02). A similar trend between nc44s2 and control, even though under statistical significance, was measured for Hthy myoblasts (Figure 5B). These findings suggest the lnc44s2 involvement during the differentiation; consequently, its potential regulatory role at three days of differentiation was studied. Then, the expression of a late differentiation myomarker, the myosin heavy chain (MyHC) was analyzed by RT-qPCR, and a significant increase of its transcription was observed in the nc44s2 condition compared to control in the 45-55 and Hthy cell line 3 days after differentiation (*p* = 0.03) (Figure 5C). In addition, the fusion index analysis at the same timepoint revealed a significantly increased differentiation rate in the 45-55 nc44s2 condition compared to control (*p* < 0.004) (Figure 5D, right panel).

**Figure 6:**
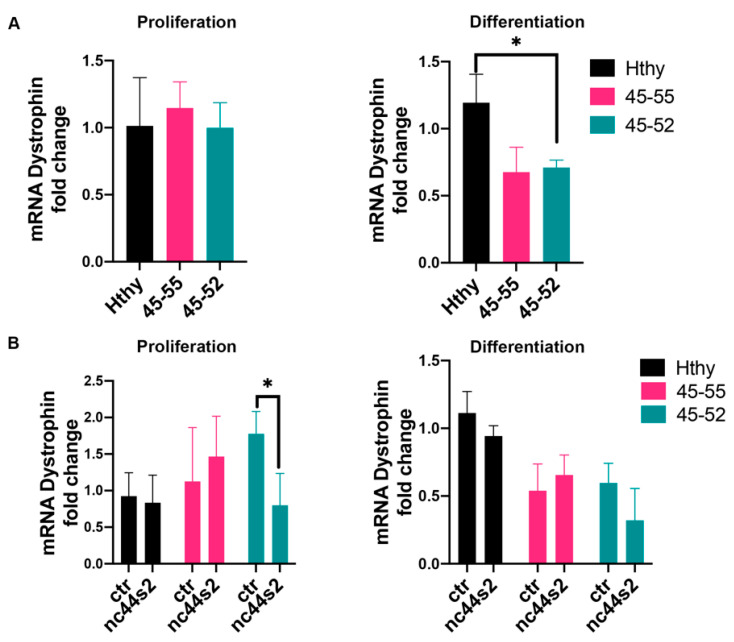
Dystrophin mRNA expression assessment. (**A**) mRNA dystrophin expression assessed by RT-qPCR at 3 days of proliferation (**left**) and at 3 days of differentiation (**right**) in human primary myoblasts issued from healthy subjects (Hthy), BMDΔ45-55 patients (45-55), and DMDΔ45-52 patient (45-52) from three independent experiments, performed in duplicates; * *p* = 0.01 by one-way ANOVA (Means ± SD). (**B**) mRNA dystrophin expression after lnc44s2 OE in the same cell lines at 3 days of proliferation and differentiation from three independent experiments, performed in duplicates; *p* = 0.01 by two-way ANOVA (Means ± SD). Abbreviations: ctr = AAV9-control condition, nc44s2 = AAV9-44s2-lncRNA OE.

lncRNA from introns 44 and 55 were described as downregulating the full-length mRNA dystrophin expression in both in vitro experiments and DMD female carriers [37]. Thus, we analyzed the mRNA level of dystrophin expression by RT-qPCR during proliferation and differentiation. DMD myoblasts did not express dystrophin protein, whereas 45-55 and 45-52 human primary myoblasts expressed significantly lower dystrophin transcripts compared to Hthy three days after differentiation (ctr/45-55: *p* < 0.002, ctr/45-52: *p* < 0.005) (Figure 6A).

To further establish if lncRNA44s2 could affect normal and dystrophic muscle cells behavior, we measured dystrophin expression in the OE experiment. At three days of proliferation, 45-52 nc44s2 OE showed reduced dystrophin transcription OE compared to 45-52 control (Figure 6B, left). A similar trend for both 45-52 and control myoblasts was observed 3 days after differentiation (Figure 6B, right panel).

The reported results show that lncRNA44s2 OE induced a decreased proliferative rate in all 3 primary myoblasts, an increased expression of differentiation myomarkers at three days of differentiation in 45-55, and an increased fusion index. Altogether, these data suggest a potential role for lncRNA44s2 during myogenic differentiation, which could participate in BMD pathophysiology.

## 4. Discussion

In this translational study we addressed the question of the role of lncRNAs in BMD phenotype variability based on clinical phenotype characterization, in silico breakpoint analysis of BMDΔ45-55 patients, and in vitro experiments in dystrophic and control myoblasts. We selected 4 lncRNA bordering the in-frame exons 45 to 55 deletion mutation in the *DMD* gene of BMDΔ45-55 patients. Previous work described BMD patients with 45-55 exonic deletions with a favorable clinical phenotype [30,38,39]. We completed these data with clinical characterization of skeletal muscle impairment in 38 BMDΔ45-55 patients according to a modified clinical severity scale (CSS). Here, we presented in silico analysis of the breakpoint of the deletion (±10 bp) in diagnostic DNA of 18 patients BMDΔ45-55 based on WGS data.

Miyazaki et al. studied the deletion breakpoint in three BMDΔ45-55 patients and identified specific breakpoints for each patient but did not identify any significant homology between the distant and proximal sequences [38]. In this report, the WGS analysis of diagnostic DNA of 18 patients revealed that only family cases had a similar breakpoint and that there were two most frequent breakpoint sites (±10 bp). Interestingly, only one patient had a very large deletion of 200 kb. This particular feature of the intron 44 and 55 might be explained by the large size of the introns [40]. Furthermore, several previous studies identified a deletion hotspot in the *DMD* gene involving exons 45-55 [23,24,38,41].

The profile of the genomic presence of the 4 lncRNA in a total number of 38 patients was analyzed: 19 patients had a specific breakpoint with preserved lncRNA sequences localized in the introns 44, 11 patients preserved all lncRNA sequences, 7 patients had only lncRNA 44s, and 1 patient had no lncRNA sequences (Figure 1). These findings highlighted the presence of the two most frequent clusters: one preserving the lncRNA from intron 44 and the second preserving all lncRNAs.

In addition, a correlation of favorable mild CSS skeletal muscle phenotype and unique presence of lncRNA44s on the genomic level was identified. All seven patients from the first cluster had a mean age of 37 ± 20 years old, and none had a severe CSS phenotype but only mild and intermediate CSS. Taking into consideration the mean age of the first cluster, it can be considered that those patients had a high chance of remaining in a mild CSS state, as their disease severity was assessed at an age similar to when supposed muscular deficits are displayed (mean age at 37 ± 19 years old). In the second cluster, with present lncRNA44s and 44s2, the patient mean age was 39 ± 19 years with mild and intermediate CSS. Despite the presence of one severe CSS patient who lost ambulation at 72 years old but had a mild deficit in the upper limb and was autonomous for wheelchair transfers. Altogether, this information suggested a trend to less severe phenotype in patients who preserved the lncRNA localized in intron 44. In addition, the expression levels of lncRNA 44s and 44s2 were similar between control and BMDΔ45-55 muscular biopsies and, in contrary, had significantly lower expression in severe BMDΔ3-7, suggesting a positive correlation with the less severe phenotype.

In accordance with published data [11,12], in vitro lnc44s2 OE study in human primary myoblasts of Hthy, 45-55, and 45-52 suggested its role in the differentiation acceleration.

Recent studies reported Linc-MD1 (long intergenic non-protein coding RNA, muscle differentiation 1) as specifically activated during differentiation in myoblasts and satellite cells [17,19,42]. Interestingly, Linc-MD1 was described as expressed in newly regenerating fiber and abundant in dystrophic condition with an expression profile similar to lncRNA 44s, 44s2, 55s, and 55as in human primary and immortalized myoblasts of BMDΔ45-55 patients described in this work. Upstream of the MyoD locus is localized the MUNC lncRNA (MyoD upstream noncoding RNA), which initiates transcription in the DRR (distal regulatory region) RNA locus [43,44]. Overexpression and downregulation experiments of MUNC in myoblasts resulted in the increase/decrease of myogenin and MyH3 (myosin 3) [43]. In a similar way, based on our findings, lncRNA 44s2 OE increased MyoD expression at 3 days of proliferation, suggesting a role of this lncRNA in the induction of myogenic commitment. Indeed, the increase of MyHC in lncRNA 44s2 overexpressing myotubes 3 days after differentiation indicates an effect of this factor in late myogenic differentiation.

Bovolenta et al. reported that lncRNA OE from intron 44 and 55 inhibited the activation of dystrophin promoter in human rhabdomyosarcoma and neuronal cells [37]. Interestingly, a previous report detected a significant heterogeneity in dystrophin expression, which varied from 50% to 80%, in BMDΔ45-55 patients [45]. Anthony et al. underlined in his study describing several DMD mutations, including del45-55 patients, not only a variability in the BMD population but also a positive correlation between dystrophin protein expression and clinical severity. The BMDΔ45-55 patients reported in this work displayed a range of 60% to 90% of dystrophin expression, and the clinical classification was mild for all four of them [46].

Current therapeutic dogma is to convert severe phenotype, such as DMD patients into milder BMD-like phenotype patients [47,48]. Actual AON-approved therapies are limited to one exon skipping. To improve such a therapeutic strategy, ongoing preclinical efforts are performing multi-exon skipping [40,41,49,50]. The complete multi-exon skipping combined with a treatment with up to 10 AON of the large, deleted exon 45-55 BMD patients revealed significant technical and practical hurdles in developing this kind of therapy [17,19,20,39,42]. On the contrary, for the limited number of eligible DMD patients and continuous lifetime administration of AON, genome editing would be able to make permanent changes to the *DMD* gene sequence [27,51,52,53]. Young et al. successfully corrected three DMD hiPSC by CRISPR-Cas9 induced NHEJ repair of the *DMD* gene. The localization of sgRNA did not preserve any of the lncRNA sequences localized in intron 44 and 55 (approximately 500 bp close to exon 44 and exon 56) [54,55]. Noteworthy, we identified only one patient with a similar very large, spontaneous *DMD* gene deletion. As the therapeutic relevance of MES45-55 could be deduced from the clinical status of BMDΔ45-55 patients carrying spontaneous deletion, we suggest reproducing the reported breakpoints during the design of chimeric introns by MES.

**Figure 7:**
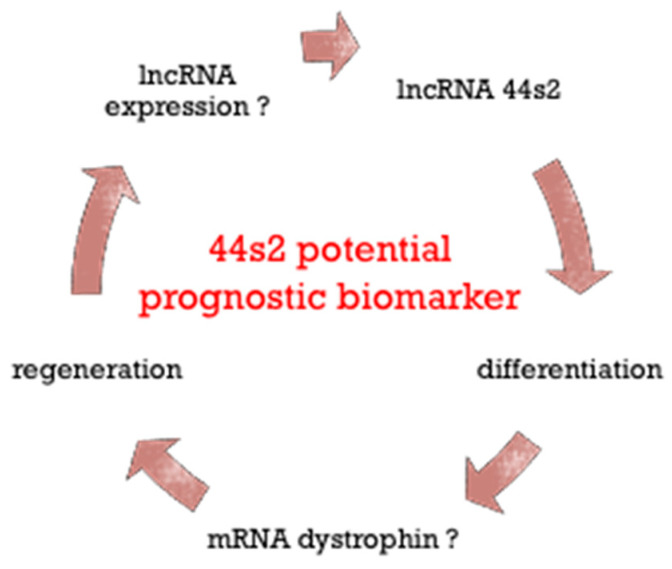
Schematic representation of lncRNA 44s2 role during regeneration in dystrophic skeletal muscle.

These in vitro study results in human primary myoblasts from BMDΔ45-55 patients reveal a possible involvement of lncRNA sequences localized in intron 44 and 55 in myogenesis, which could be interpreted as an indicator of regeneration. It can be hypothesized that lncRNA could play a key role in dystrophic muscles to alleviate the disease. The lncRNA expression in the BMDΔ45-55 patients having a mild phenotype was similar to a healthy subject, while lncRNA was less expressed in DMD patient muscular biopsies. These data suggest that the expression of lncRNA 44s2 could be associated with a favorable outcome reflecting the regeneration process (Figure 7).

Overall, these findings suggest that lncRNA 44s2 could be involved in the muscle differentiation process and potentially become a disease progression biomarker.

## Data Availability

Data available on request due to restrictions eg privacy or ethical. The data presented in this study are available on request from the corresponding author. The data are not publicly available due to patient medical information.

## Supplementary Materials

The following are available online at https://www.mdpi.com/2227-9059/9/2/219/s1, Figure S1: OE nc44s2 evaluation in human primary myoblasts. Table S1: List of primers.
