# Supplementary material for: The lncRNA 44s2 Study Applicability to the Design of 45-55 Exon Skipping Therapeutic Strategy for DMD
Source: Biomedicines. 2021 Feb 20;9(2):219. doi: 10.3390/biomedicines9020219 (PMC7924625; doi:10.3390/biomedicines9020219)
Supplement: Supplementary file 1 [file biomedicines-09-00219-s001.pdf]

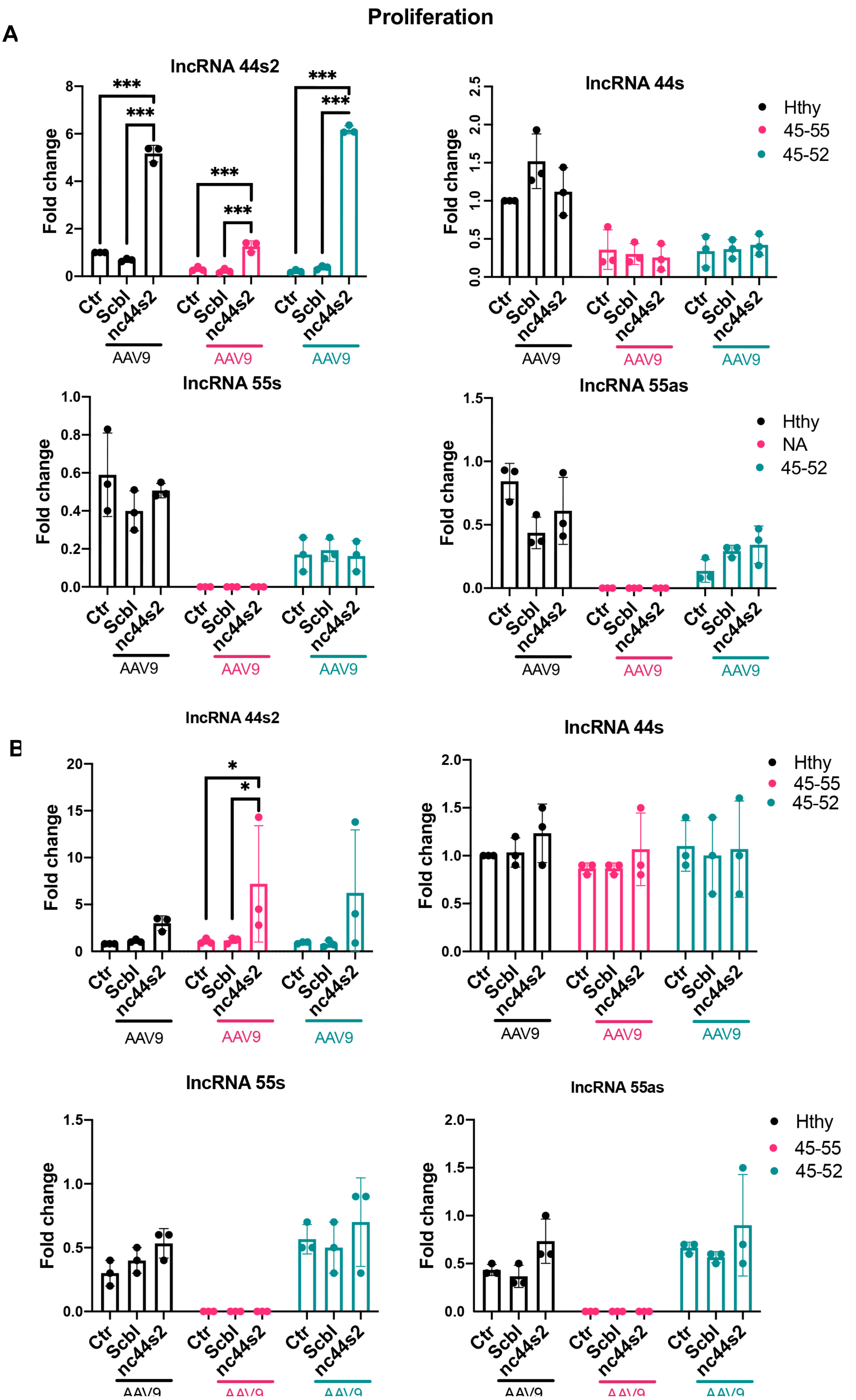

*Figure S1. OE nc44s2 evaluation in human primary myoblasts from healthy subject (Hthy, black columns), BMDdel45-55 patient (45-55, pink columns) and DMDdel45-52(45-52, green columns) during proliferation (A) and differentiation (B) Means ± SEM, from three independent experiments; *P<0.05, **P<0.01, and ***P<0.001 by two-way ANOVA test).*

pplementary Data

Name	Sequences	Tm	Amplicon length
ncINT44s_F	GTTGAGTGGGGCAATTTCTG	55 °C	531bp
ncINT44s_R	GTCACCCTGAATGACCTTCC		
ncINT44s2_F	ACCAGGAGCTCTGCTTGCAT	55°C	71bp
ncINT44s2_R	TTGTGCATGATAATGTGCCTCAA		
ncINT55s_F	TGATAACTTTCATGCCCATTAACATAG	50°C	68bp
ncINT55s_R	AACAGGACACAAATTCAGCACTTC		
ncINT55s_F2	AGCTCTGTGAAGAGACGGGAAG	55°C	836bp
ncINT55s_R2	CTGGAGAAAGGAGCCAGTGA		
ncINT55as_F	CATTTAGAGCAAGAGATACAGGCATT	55°C	761bp
ncINT55as_R	CCAGCATTTTGCTTCGGAGG		
huPO_F	GGCGACCTGGAAGTCCAACT	55°C	149bp
huPO_R	CCATCAGCACCACAGCCTTC		

Table 1Primers list for
